# A high-throughput, streamlined cloning protocol to generate guide RNAs for CRISPR activation 

**DOI:** 10.3389/fgeed.2026.1787203

**Published:** 2026-05-20

**Authors:** Shusen Zhu, Aura A. Tamez González, Abdelrahman Alokda, Jeremy M. Van Raamsdonk

**Affiliations:** 1 Department of Neurology and Neurosurgery, McGill University, Montreal, QC, Canada; 2 Metabolic Disorders and Complications Program, and Brain Repair and Integrative Neuroscience Program, Research Institute of the McGill University Health Centre, Montreal, QC, Canada; 3 Division of Experimental Medicine, Department of Medicine, McGill University, Montreal, QC, Canada

**Keywords:** *C. elegans*, cloning, CRISPR activation, guide RNA, high-throughput screening

## Abstract

Caenorhabditis elegans is a powerful model for studying gene function and disease pathogenesis. While RNA interference effectively suppresses gene expression, CRISPR activation (CRISPRa) provides a robust tool for upregulating endogenous gene expression in *C. elegans*. Compared with traditional injection-based methods, feeding-based CRISPRa, similar to RNA interference, is easy, cost-effective and efficient. However, the traditional cloning workflows remain a bottleneck for high-throughput gene screening. Here, we present a pooled, one-step dual gRNA cloning protocol that enables rapid and efficient construction of gRNA expression vectors for CRISPRa. In a test case, by designing 55-mer and 54-mer primers, each containing one gRNA, we amplified and pooled 126 gRNA inserts in a single reaction pipeline. The 126 gRNA clones were completed in three pooled rounds, achieving 42%–56% coverage for each round, with remaining clones processed individually. This protocol dramatically reduces time, labor, and reagent consumption, while increasing scalability and maintaining reproducibility. It is particularly well suited for high-throughput screening of gene libraries or pathways and supports downstream applications such as phenotypic screening and lifespan analysis. This work advances CRISPRa-based functional genomics in *C. elegans* by providing a practical tool for large-scale gene activation studies.

## Graphical Abstract

**Figure:**
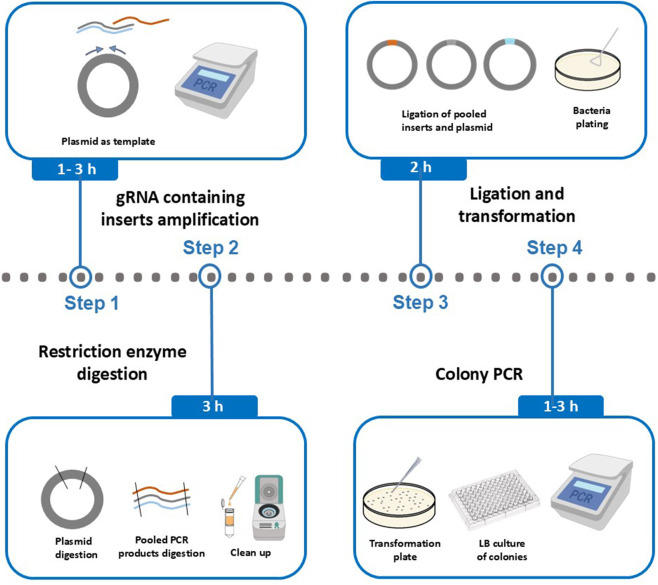

## Introduction

*Caenorhabditis elegans* has long served as a powerful model organism for studying gene function, metabolic pathways and understanding the genetic basis of development, neurobiology, and aging. RNA interference (RNAi) has played a central role in functional genomics by enabling systematic gene knockdown. In *C. elegans*, RNAi can be easily achieved by feeding worms with bacteria expressing double-stranded RNA targeting the gene of interest. This approach also enables temporal control of gene silencing, as genes can be knocked down for specific periods of time during development or adulthood by transferring the worms on and off the RNAi bacteria. Furthermore, tissue-specific knockdown can be achieved using specially constructed tissue-specific RNAi strains in which necessary components of the RNAi machinery are expressed under tissue-specific promoters in a mutant that is globally deficient in that specific component (Timmons et al., 2003; Zou et al., 2019).

However, RNAi is limited to gene silencing and thus not useful in examining the effect of gene activation or gain-of-function effects. For instance, genes involved in energy metabolism and the endoplasmic reticulum unfolded protein response (ER-UPR) are downregulated with age (Roux et al., 2023), and reduced expression of these genes is implicated in aging and neurodegenerative diseases (Lindholm et al., 2006; Li et al., 2020). Upregulating or overexpressing these genes could provide insights into the mechanisms of aging, the pathogenesis of neurodegenerative diseases and their potential protective or restorative functions. Currently, increasing gene expression in *C. elegans* typically requires generating a transgenic strain through microinjection (Wang et al., 2023) or microparticle bombardment (Schweinsberg and Grant, 2013). These methods are labor-intensive, time-consuming, less efficient, and can cause unintended genetic disruptions due to random integration.

A practical alternative for gene upregulation is CRISPR activation (CRISPRa), a technique that uses a modified CRISPR-Cas9 system to enhance the expression of specific genes without altering the underlying DNA sequence. CRISPRa uses a deactivated Cas9 protein (dCas9), which binds to specific DNA sequences, without cleaving the DNA. To enhance gene activation, dCas9 is fused to a transcriptional activator domain, such as VP64, p65 or p300, which recruit the cell’s transcription machinery. The dCas9-activator complex is then directed by a guide RNA (gRNA) to the target gene’s promoter region to boost transcription. To achieve this, the gRNA is composed by a scaffold sequence that will bind to the dCas9, and a guide sequence complementary to the target. This method allows precise, reversible control of gene activity and is widely used in disease modeling, drug discovery, and functional genomics (Maeder et al., 2013; Chavez et al., 2016; Clement et al., 2020).

In *C. elegans*, CRISPRa has been adapted to activate genes by delivering promoter-specific gRNAs *via* bacterial feeding, as demonstrated by Fischer et al. (2022). This method uses the L4440_BioBrick-sgRNA vector to express a pair of gRNAs in HT115 *E. coli* bacteria, which is then fed to a *C. elegans* strain expressing a codon optimized dCas9::VP64 fusion protein to activate endogenous genes. The pair of gRNAs act synergistically to enhance transcriptional activation. While this feeding-based system simplifies gRNA delivery, is effective for individual gene studies and can achieve similar results to conventional injection-based methods, the gRNA cloning process for large-scale or genome-wide screens remains a bottleneck due to its time-consuming multi-step and labor-intensive nature.

To address this limitation, we developed a streamlined, one-step CRISPR_Cas9 dual gRNA cloning protocol combined with a pooling strategy to generate gRNA expression vectors for high-throughput CRISPRa screening in *C. elegans*. By designing two primers containing the gRNA common sequences, using the same vector for cloning as template to amplify cloning inserts, modifying the cloning method from two-step to one-step, and pooling the cloning inserts into a single sample, our approach can simultaneously generate gRNA constructs targeting hundreds or even thousands of genes. This method significantly reduces time, labor, and reagent costs, making it ideal for large-scale gene activation studies, such as those targeting gene libraries or pathways, and supports downstream applications like phenotypic screening and lifespan analysis in *C. elegans*.

## Materials

### Reagents

Platinum™ SuperFi™ II DNA Polymerase (ThermoFisher #12361010)

10 mM dNTPs (BaoBasic #DD0056)

Monarch PCR and DNA Cleanup Kit (NEB #T10306)

QIAquick and Gel Cleanup kit (Qiagen #28506)

EZ-10 Spin Column Plasmid DNA Minipreps Kit (BioBasic #BS6149)

BsaI-HF (NEB #R3733S)

BbsI-HF (NEB #R3539S)

Antarctic Phosphatase (NEB ##M0289)

Instant Sticky-end Ligase Master Mix (NEB #M0370S)

HT115 competent cells (self-made)

2 × Rapid Taq Master Mix (Vazyme #P222-02)

IPTG (GOLDBIO #I2481C100)

Carbenicillin Disodium (GOLDBIO #C-103-50)

Tetracycline Hydrochloride (GOLDBIO #T-101-100)

## Methods

**FIGURE 1:**
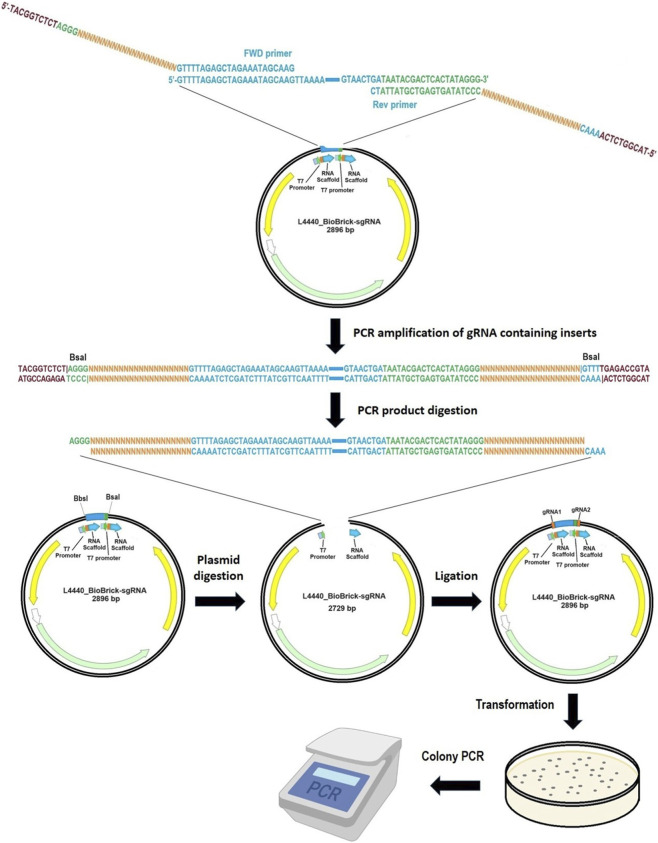
Workflow of this cloning protocol. Step-by-step schematic describing the protocol. The same plasmid serves as both a template for amplifying the gRNA-containing insert and as an expression vector. From gRNA-containing inserts amplification to colony PCR ready for sequencing takes 2–3 days. This figure was created with BioRender.com.

An overview of the workflow for this protocol is presented in Figure 1.

### Clone specific primer design

1. Select the gRNA target sequences from the promoter region of your target gene. Check the gRNA sequences to ensure that they do not contain BsaI restriction sites, as this enzyme will be used for digestion.

Note: We selected gRNAs from the library generated by Fischer et al. (Fischer et al., 2022) and double checked with IDT online tool “CRISPR-Cas9 gRNA checker” to identify the ones with both on-target and off-target scores over 50, the higher the better. For genes not covered by the library, we located the transcription start site (TSS) of the gene by looking up previously published data (Chen et al., 2013; Kruesi et al., 2013; Saito et al., 2013) and then located the region −50 to −400 bp upstream of the TSS (Gilbert et al., 2014). Using that sequence, we designed the gRNAs using the IDT online tool “Design custom gRNA”, which generates 20 nt long gRNAs that are adjacent to the NGG PAM motif (Gilbert et al., 2014). Since this protocol uses a dual gRNA expression vector, at least two gRNAs should be selected for each gene. We designed four gRNAs targeting different and dispersed sites for optimizing gene expression as gRNAs targeting different sites may have varying efficiencies and specificities. Therefore, two constructs for each gene were generated.

2. Design the primers with BsaI restriction sites as below, replacing the 20 Ns with your gRNA target sequences.

Forward primer

5′-TACGGTCTCTAGGGNNNNNNNNNNNNNNNNNNNNGTTTTAGAGCTAGAAATAGCAAG-3′

Reverse primer

5′-TACGGTCTCAAAACNNNNNNNNNNNNNNNNNNNNCCCTATAGTGAGTCGTATTATC-3′

Note: These primers are used to amplify the cloning insert including the first gRNA scaffold and the second T7 promoter between the two restriction sites. Be careful to replace the 20 Ns in the reverse primer with the reverse complement sequence of your second gRNA target sequence.

Note: We added BsaI cut sites in both primers because: 1) this makes the digestion with single enzymes easier; 2) BsaI is much less expensive than BbsI; 3) PCR products have no DNA methylation and BsaI digestion will not be interfered; and 4) BsaI is a Type II restriction enzyme, and its overhangs are compatible with BbsI.

### Cloning inserts preparation

3. Use the designed primers and the L4440_Biobrick EV template to PCR-amplify the cloning inserts.

PCR reaction mixture for one insert.

**Table:** 

Reagent	Amount (µL)
5X SuperFi II buffer	4
10 mM primer FR mix	0.8
10 mM dNTP	0.4
plasmid L4440_BioBrick-EV template	1 (1 ng)
SuperFi II DNA polymerase	0.4
Nuclease-free water	13.4
Total	20

PCR program

**Table:** 

Steps	Temperature	Time	Cycles
Initial denaturation	98 °C	30 s	1
Denaturation	98 °C	10 s	28
Annealing	60 °C	10 s	
Extension	72 °C	5 s	
Final extension	72 °C	2 min	1
Hold	4 °C	∞	1

Note: Run all reactions in 96-well PCR plates.

4. Run 2 µL of each PCR product on a 2% agarose gel to check the size and the yield of the PCR product. All the PCR products should give a strong single band of 191 bp in size.
5. Take equal amounts (1–3 µL) of each PCR product and pool them in a single tube. Purify the pooled PCR products using PCR clean-up columns following the manufacturer’s instructions. Measure the DNA concentration using a Nanodrop Microvolume Spectrophotometer.

Note: Hundreds or thousands of PCR products can be pooled and mixed equally as the primer lengths, primer binding sequences, PCR conditions, and the length of PCR products for different reactions are all the same. Thus, the PCR efficiencies and the yields should be the same as well. These PCR products need to be amplified only once for pooled cloning strategy. The remaining PCR products can be saved at −20 °C and used later for multiple rounds of cloning.

6. Digest the purified PCR products with BsaI-HF.

Prepare the digestion reaction.

**Table:** 

Reagent	Amount (µL)
Purified PCR products	X (1 µg)
10X CutSmart buffer	5
BsaI-HF	1
Nuclease-free water	44 - X
Total	50

Incubate the reaction at 37 °C for 1 h, and then inactivate the enzyme at 80 °C for 20 min.

Note: Digestion of 1 µg of the purified PCR products is enough for the following ligation. This digestion will generate a mix of 167-bp fragments with sticky ends ready for ligation:

5′-AGGGNNNNNNNNNNNNNNNNNNNNGTTTTAGAGCTAGAAATAGCAAGTTAAAATAAGGCTAGTCCGTTATCAACTTGAAAAAGTGGCACCGAGTCGGTGCTTTTTTTAGGTTCTGTTAAGTAACTGATAATACGACTCACTATAGGGNNNNNNNNNNNNNNNNNNNN-3′

The sticky end of the double strands of the DNA fragments after digestion:

5′-AGGGNNNNNNNNNNNNNNNNNNGTT … … AGGGNNNNNNNNNNNNNNNNNN-3′

3′-NNNNNNNNNNNNNNNNNNCAA … … TCCCNNNNNNNNNNNNNNNNNNCAAA-5′

7. Purify the digested PCR products using PCR clean-up columns following the manufacturer’s instructions, same as step 5. Measure the DNA concentration by Nanodrop Microvolume Spectrophotometer.

Note: You may skip this purification step if NEB Instant Sticky-end Ligase Master Mix is applied and you are doing a ligation for a single clone without the need of many colonies as this ligation reagent is very efficient. However, purification is strongly recommended for the pooled cloning method.

### Cloning vector preparation

**Table:** 

Reagent	Amount (µL)
Plasmid DNA	X (4 µg)
10X CutSmart buffer	10
BsaI-HF	2
BbsI-HF	2
Nuclease-free water	86 - X
Total	100

Incubate the reaction at 37 °C for 1 h. Then add 2 µL of Antarctic Phosphatase +11.3 µL of 10X Antarctic Phosphatase reaction buffer and continue the incubation at 37 °C for another 30 min. Heat the reaction at 80 °C for 20 min to inactivate the enzyme.

Note: This digestion will generate four fragments of size 2729-bp, 119-bp and two 24-bp. The dephosphorylation step could be omitted, but without dephosphorylation, the transformation could lead to more false positive colonies as BsaI-HF, used for this experiment is dcm methylation sensitive which may result in incomplete digestion. 4 μg of digested plasmid is enough for five hundred clones.

8. Digest the vector L4440_BioBrick-sgRNA with BbsI + BsaI and dephosphorylate the digested vector to prevent self-ligation.
9. To purify the digested plasmid, load all the reaction mixture and loading buffer into a big well of an agarose gel, run a gel electrophoresis and perform a gel extraction. For the extraction, the bands on the gel are visualized in an open UV box, the 2729-bp fragment is excised using a scalpel, and then this fragment is isolated from the gel using a DNA purification kit following the manufacturer’s instructions

Note: Purification of digested plasmids can be done by column clean-up, while gel extraction is recommended as this extraction can improve transformation efficiency. This step only needs to be done once, and the purified plasmids can be used for multiple rounds of cloning. If column clean-up is applied, the 119-bp fragment which has the same sticky end as the vector can be retained in the purified vector, competing with the vector and ligate with the digested PCR products in the following ligation reaction. In this case, the amount of the inserts used for ligation should be double.

### Ligation and transformation

10. Perform ligation with the digested, purified PCR products and plasmid at molar ratio of 4:1. Apply 50–100 ng of plasmid for each ligation reaction.

**Table:** 

Reagent	Amount (µL)
Instant sticky-end ligase master mix	5
Plasmid DNA	X (50–100 ng)
Pooled and purified PCR products	Y (12–24 ng)
Sterile water	5-X-Y
Total	10

Add 5 μL of Instant Sticky-end Ligase Master Mix to the 5 μL of the combined plasmid and inserts, mix thoroughly by pipetting up and down 7-10 times, and place on ice.

11. Perform transformation of HT115 competent cells with the ligation mix. Thaw 50–100 μL of HT115 competent cells in a 1.5-mL tube on ice. Add 5 μL of the ligation mix to the cells and mix by finger-flicking. Incubate the tube on ice for 30 min. Heat shock at 42 °C for 1 min, return the tube to ice for 5 min. Add 700 μL of SOC medium without antibiotics to the tube and incubate for 1 hour at 37 °C with shaking.
12. Spread 100 μL of the non-concentrated culture on one LB plate containing carbenicillin and tetracycline, and then concentrate the rest by centrifugation to 100 μL and spread on another one or two plates. Incubate at 37 °C overnight.

Note: Since the transformation efficiency of HT115 competent cells is much lower than that of DH5α competent cells, different amounts of cells should be tried in the first cloning to get a plate with appropriate number of colonies.

### Colony PCR screening

13. Pick up single colonies and grow each colony in 50 μL of LB medium containing 50 μg/mL carbenicillin in 96-well culture plates at 37 °C for 1–2 h.
14. Take 0.8 μL of each bacterial culture as template to run a PCR using the three primers listed in Table 1 to screen for positive colonies.

**TABLE 1:** Primers used in this protocol.

Primers	Sequences (5′ →3′)	Optimal TA (°C)
Cloning inserts	F: TACGGTCTCTAGGGNNNNNNNNNNNNNNNNNNGTTTTAGAGCTAGAAATAGCAAG R: TACGGTCTCAAAACNNNNNNNNNNNNNNNNNNCCCTATAGTGAGTCGTATTATC	60
Colony PCR for screening	F: AGGCGATTAAGTTGGGTAAC R1: AGAGACCTACACTGGTCTCT R2: AACCTGGCTTATCGAAATCT	57
Colony PCR for sequencing	CAAGTGTAGCGGTCACGCTG GTGAGCGAGGAAGCAACCTG	66

Prepare PCR reaction mixture for one colony.

**Table:** 

Reagent	Amount (µL)
2x Rapid taq master mix	5
Primer mix	0.4
Bacteria culture	0.8
Nuclease-free water	3.8
Total	10

Note: The final concentration of the three primers is 400 nM of F, 400 nM of R1 and 80 nM of R2 respectively.

**FIGURE 2:**
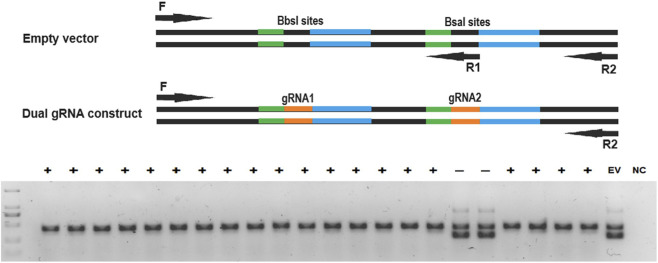
Colony PCR screening. Colony PCR using three primers with one internal primer R1 that only binds to the empty vector specific sequence at the BsaI cut sites produces two bands with sizes of 277 bp and 419 bp for a negative colony and a 419-bp single band for a positive colony. F: forward primer; R1: internal reverse primer; R2: external reverse primer; +: positive colony, -: negative colony, EV: empty vector, NC: PCR negative control.

15. Check the PCR products on a 2% agarose gel. A positive colony should show a 419-bp strong single band, and a negative colony transformed with an empty vector should show two bands with sizes of 277 bp and 419 bp (Figure 2).

Run PCR program

**Table:** 

Steps	Temperature	Time	Cycles
Initial denaturation	95 °C	5 min	1
Denaturation	95 °C	15 s	30
Annealing	57 °C	15 s	
Extension	72 °C	7 s	
Final extension	72 °C	2 min	1
Hold	4 °C	∞	1

Note: You can skip this step if you are cloning a large number of gRNAs using the pooled method as the positive rate is very high. In our case >98% of the colonies are positive. You can run a colony PCR using the bacterial cultures as templates and another set of primers for sequencing (see the table) and directly send the non-purified PCR products for sequencing.

16. (Optional) Double check to confirm your clones after sequencing by colony PCR using the gRNA containing primers, See Table 1.
17. Check the PCR products on a 2% agarose gel. Correct clones should show a strong single band with 191 bp in size.

Note: This step is not necessary but can be used to confirm that there were no mix ups in going from the 96 well plate to sequencing and back to the 96 well plate.

Prepare PCR reaction mixture for one clone.

**Table:** 

Reagent	Amount (µL)
2x Rapid taq master mix	5
10 mM primer mix	0.2
Bacteria culture	0.8
Nuclease-free water	4
Total	10

PCR program

**Table:** 

Steps	Temperature	Time	Cycles
Initial denaturation	95 °C	5 min	1
Denaturation	95 °C	20 s	30
Annealing and extension	73 °C	15 s	
Final extension	72 °C	2 min	1
Hold	4 °C	∞	1

### Sequencing-based validation of gRNA inserts

For sequencing-based validation of the gRNA inserts we used a reverse primer 142 bp downstream of the 3′cloning site. The sequence of the primer utilized was: AAC​CTG​GCT​TAT​CGA​AAT​CT. Using this primer, we typically obtained clear sequencing reads from about 50 bp downstream of the 3′cloning site to 265 bp upstream of the 5′cloning site. Sequencing reads were analyzed to confirm the correct full-length insert and correct orientation. After sequencing all of the clones that were picked in each round of cloning, the obtained sequences were compared to the known sequences of the target genes.

## Anticipated results

The colony distribution of different clones should be uniform, and the chance of getting any specific clone should be equal to getting any other clone. The number of colonies that need to be screened to obtain a specific number of clones can be calculated according to the formula:

$$E\left(m,n\right)=\sum_{k=0}^{m-1}\frac{n}{n-k}$$

In this formula E (m, n) represents the expected number of colonies that need to be picked to obtain at least m different clones from n uniformly mixed clones (m < n). For instance, suppose we have n = 100 clones and try to obtain *m* = 60 different clones for the first round cloning, then we calculate the expected number of colonies we need to pick up for screening: E (60,100) = $\sum_{k=0}^{59}\frac{100}{100-k}$ = $\frac{100}{100}+\frac{100}{99}+\frac{100}{98}+\ldots +\frac{100}{42}+\frac{100}{41}$ = 90.88. That means at least 91 colonies need to be screened to obtain 60 different clones. Then for the second-round cloning, we will calculate the number of colonies needed to be screened for the remaining n = 40 clones. This calculation is easy to perform in an Excel file (Refer to the calculation examples in the Supplementary Excel File). According to this formula, the fewer the number of colonies screened, the higher the ratio of obtaining different clones. Although it is possible to obtain all different clones through one round of cloning, that is, let m = n, the number of clones that need to be screened would be huge. In the example above, if m = n = 100, at least 519 colonies need to be screened to get the 100 clones by doing one round cloning. Therefore, cloning should be performed in multiple rounds to optimize efficiency.

When the number of remaining clones is less than 15, we recommend generating each clone individually in the last round of cloning as it is very manageable to process this number of clones at once. However, based on our experience, the actual clonal coverage is always lower than the theoretically predicted coverage due to sequencing failures, false positives, and mutations in some samples. Another reason could be that the number of colonies containing different constructs may not be equal nor evenly distributed since the expression of different gRNAs may have different impacts on colony growth.

**FIGURE 3:**
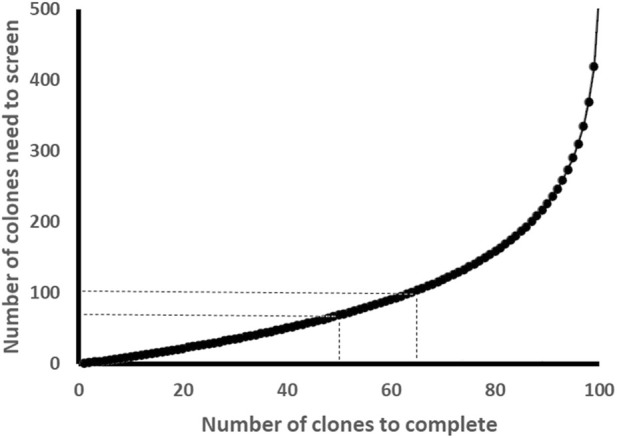
The relationship between the number of screened colonies and the number of obtained clones. Theoretically, the fewer colonies screened, the higher the probability of obtaining different clones-in other words, the higher the screening efficiency. While, the fewer colonies screened, the more cloning cycles are required. To balance and minimize the number of colonies screened and the number of cloning cycles, the optimal number of colonies screened in each cloning cycle should be approximately 70%–100% of the total number of clones. This theoretically yields 50%–65% of clones, but in practice, about 45%–57% are obtained.

In summary, when the total number (n) of clones is fewer than 500, screening colonies equivalent to 70%–100% of n during each round of pooled cloning is generally effective, and theoretically, about 50%–65% of n clones can be obtained (Figure 3). Considering the actual number of clones obtained is less than the theoretically calculated number, completion of all cloning is typically achieved through three to four rounds of pooled cloning, followed by a final round of individual cloning. Overall, the total number of colonies requiring screening is approximately 1.5n to 1.8n. For larger-scale efforts involving more than 1,000 clones, to avoid screening excessively large numbers of colonies in a single round, it is preferable to perform additional rounds of pooled cloning while initially screening only 30%–50% of n. This strategy tends to improve the yield per screening round, ultimately reducing the total number of colonies screened to below 1.5n and enhancing overall cloning efficiency.

Each round of cloning takes only 2–3 days. As PCR amplification, vector digestion, dephosphorylation and gel extraction need to be done only once, only the first round of cloning from PCR amplification to transformation takes longer time (approximately 2 days). All the following steps, such as pooled PCR product purification, digestion, purification again, ligation and transformation, for the subsequent rounds of cloning can be completed in 1 day. The next day, colony PCR can be performed for screening and sent for sequencing. The process from cloning to getting sequencing results can be completed within 1 week.

We have used this protocol to generate selected 126 gRNAs for our studies on the biology of aging. Cloning was performed in three rounds using the pooled strategy (see Table 2 for summary). The average colony positive rate was about 98%. In the first round, primers were included for 126 gRNA clones. We picked 112 colonies for sequencing. 59 of these colonies contained a unique gRNA sequence, while the remaining 53 were duplicates. In the second round, we included primer pairs for 72 gRNA clones, picked and sequenced 65 colonies and obtained 30 unique gRNA clones with 35 duplicates. Finally, in round 3, we included 45 primer pairs, picked and sequenced 45 colonies and obtained 25 unique gRNA clones with 20 duplicates. The remaining gRNA clones were generated manually without pooling.

**TABLE 2:** Summary of Pooled Cloning. In the first round, primers were included for 126 gRNA clones. We picked 112 colonies for sequencing. 59 of these colonies contained a unique gRNA sequence, while 53 were duplicates. In the second round, we included primer pairs for 72 gRNA clones, picked and sequenced 65 colonies and obtained 30 unique gRNA clones with 35 duplicates. Finally, in round 3, we included 45 primer pairs, picked and sequenced 45 colonies and obtained 25 unique gRNA clones with 20 duplicates. The remaining gRNA clones were cloned manually without pooling.

Number of times a specific gRNA clone was picked	Number of gRNA clones that were picked the indicated number of times		
	Round 1	Round 2	Round 3
8 times	0	1	1
7 times	0	0	0
6 times	1	0	0
5 times	3	2	2
4 times	4	3	0
3 times	4	3	0
2 times	16	5	5
1 time	31	16	17
0 times	67	42	20
Number of unique gRNA clones picked	59	30	25
Total number of gRNA clones picked	112	65	45
Total number of gRNA primer pairs used	126	72	45
Number of unique gRNA clones picked as a percentage of starting number of primer pairs	46.8%	41.7%	55.6%

In the first two rounds of cloning, we found that 22 of the 177 clones sequenced exhibited differences from the known sequence. Thirteen of these errors were point mutations that occurred in the primer region suggesting that these differences in sequence were likely caused by oligonucleotide synthesis errors in generating the 54/55-mer primers. Excluding the thirteen point mutations in the primer region, there were a total of nine mutations in the 177 clones sequenced for a mutation rate of 5%.

## Discussion

**FIGURE 4:**
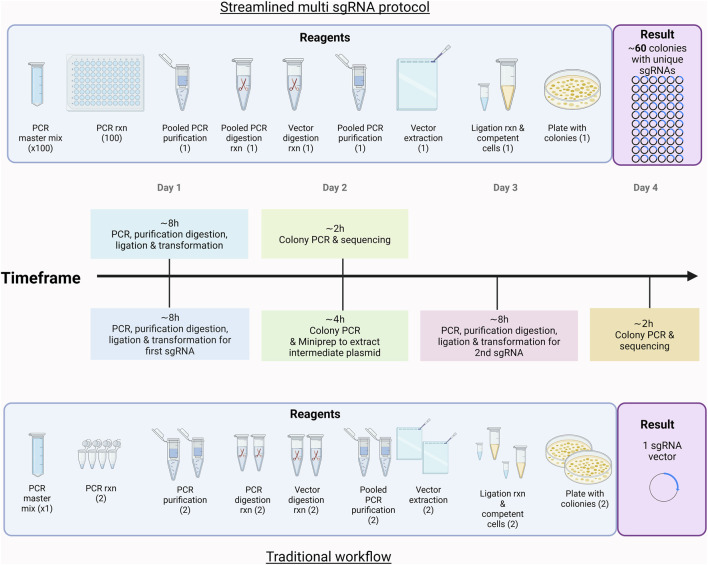
Time and reagents used in the production of a single gRNA vector compared to the streamlined multi-gRNA protocol. This figure was created with BioRender.com.

Our high-throughput, pooled cloning protocol for CRISPRa screening significantly improves the efficiency of traditional gRNA cloning methods. This efficiency stems from the uniform design of primers and PCR products, which ensures consistent amplification, digestion, ligation and transformation efficiencies across all clones. The pooled strategy minimizes the number of individual reactions, allowing hundreds or even thousands of constructs to be cloned simultaneously in a single tube, reducing both time and reagent consumption compared to traditional sequential cloning workflows, as shown in Figure 4 and summarized in Table 3.

**TABLE 3:** Comparison of pooled gRNA cloning to traditional gRNA cloning.

Feature	Traditional gRNA cloning	Pooled gRNA cloning
Dual gRNA cloning	Sequential or multi-step	Single-step
Insert preparation	Individual	Pooled
Ligation and transformation	Individual	Pooled
Scaling efficiency	Linear	Non-linear
Automation compatibility	Moderate	High

**FIGURE 5:**
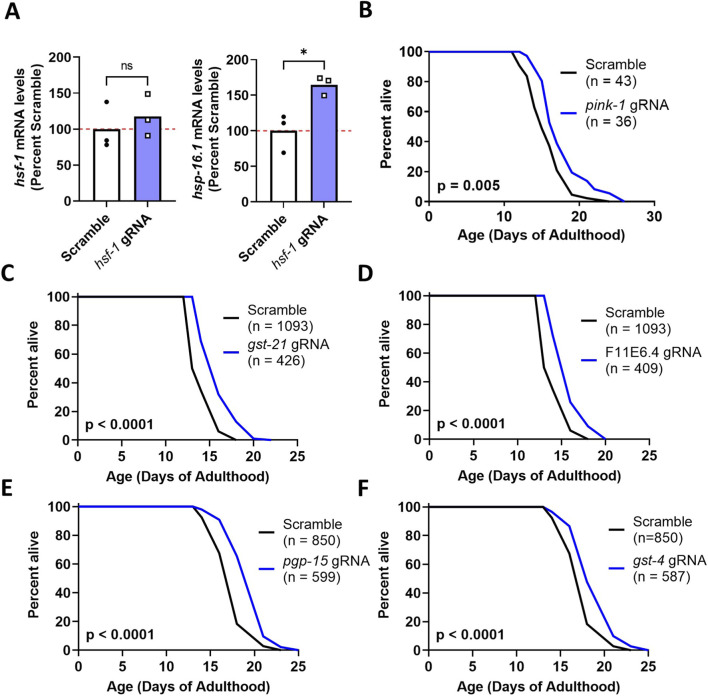
Validation of gRNA clones generated by pooled cloning method. **(A)** Worms ubiquitously expressing dCas9-VP64 treated with a guide RNA targeting *hsf-1* exhibit a trend towards increased levels of *hsf-1* mRNA and significantly increased expression of the HSF-1 target gene *hsp16.1.* gRNA treatment was initiated at the L4 stage of the parental generation and gene expression was measured in the offspring. Offspring were collected as pre-fertile young adults after life-long exposure to the gRNA. Three biological replicates were performed. Statistical significance was determined using an unpaired t-test. Worms ubiquitously expressing dCas9-VP64 treated with a guide RNA targeting *pink-1* **(B)** *, gst-15* **(C)**, F11E6.4 **(D)**, *pgp-15* **(E)** or *gst-4* **(F)** show increased lifespan compared to worms treated with a scrambled guide RNA. gRNA treatment for the lifespan study was initiated at the late L4 stage. Lifespan plates contained 50 µM 5-fluoro-2′-deoxyuridine to inhibit the development of progeny. Three biological replicates were performed. The total n number is indicated in the graphs. The Scramble control for C and D, and for E and F is the same. Statistical significance was determined using a log-rank test.

In our implementation, we achieved a 98% colony positive rate and 42%–56% clone coverage rate per round across three pooled cloning rounds for 126 gRNA clones. To determine the extent to which the gRNAs generated through this approach were able to increase gene expression, we conducted a qPCR assay to measure the mRNA levels of *hsf-1* and its downstream target *hsp16.1*, after feeding the worms with the clones containing the target gRNAs for the *hsf-1* gene. We observed a trend towards increased levels of *hsf-1* and a significant increase in *hsp-16.1* mRNA levels (Figure 5A).

To further test the function of the gRNA clones generated, some of these clones were used in a screen to find pro-longevity genes. We found that the activation of *pink-1,* which encodes a mitochondrial protein implicated in mitophagy and stress resistance, significantly extends the lifespan of *C. elegans* (Figure 5B). Furthermore, we found that activation of *gst-21,* F11E6.4, *pgp-15* and *gst-4* also significantly increase lifespan compared to a scrambled gRNA (Figures 5C–F).

The protocol’s scalability makes it particularly suited for large-scale functional genomics studies, such as screening gene libraries or entire pathways. For example, it can be applied to investigate gene networks involved in aging, stress response, or disease pathogenesis, where upregulation of multiple genes is necessary to dissect compensatory mechanisms or therapeutic targets (El-Brolosy and Stainier, 2017; Wang et al., 2022). The use of *E. coli* HT115 for gRNA delivery further simplifies the workflow, as it leverages the established RNAi feeding infrastructure in *C. elegans* research, ensuring compatibility with existing laboratory protocols (Maeder et al., 2013). Additionally, the high colony positive rate (>95%) in pooled cloning eliminates the need for extensive colony PCR screening in many cases, further streamlining the process.

As the workflow is inherently high-throughput and relies on uniform primer architecture and identical PCR product size, scaling from tens to hundreds or even thousands of gRNA constructs does not proportionally increase procedural complexity. Most of the intermediate steps—including restriction digestion, purification, ligation, and transformation—are performed on pooled samples and therefore remain essentially as simple as handling a single construct.

When scaling to larger libraries, the primary parameters that increase are (1) the number of initial PCR reactions required to generate individual inserts, (2) the total number of cloning rounds and (3) the number of colonies that need to be screened to achieve full coverage. The initial PCR amplification and colony PCR screening can be efficiently performed using multi-channel pipettes in 96-well or 384-well plate formats, substantially reducing hands-on time. For laboratories equipped with liquid-handling platforms, these steps can be further automated to enable rapid processing of large libraries.

Similarly, colony screening can be streamlined through the use of automated colony-picking systems, which markedly reduce manual labor when handling hundreds or thousands of transformants. Because the pooled ligation strategy maintains high colony positivity (>95% in our implementation), the overall screening burden remains manageable even at larger scales.

For libraries exceeding 1,000 constructs, we recommend performing additional rounds of pooled cloning while screening a smaller fraction (e.g., 30%–50%) of the total theoretical clone number per round. This strategy improves the yield-to-screening ratio and prevents excessive colony handling in any single round. Based on theoretical modeling and empirical data, the total number of colonies that must be screened generally remains within 1.5–1.8× the desired clone number, even below 1.5n at larger scales.

Collectively, because the most labor-intensive enzymatic steps are performed only once and pooled handling minimizes repetitive manipulation, the time and resource requirements don’t increase linearly relative to library size. This makes the protocol particularly well-suited for pathway-scale or genome-scale CRISPRa library construction.

This protocol can also be used for mammalian cell CRISPR-based gene activation and inhibition (CRISPRi) screening with a little modification of the expression vectors. It not only enhances the efficiency of CRISPR-based research but also makes high-throughput gene activation and inhibition screening more convenient by reducing technical and resource requirements. It enables researchers to rapidly generate gRNA libraries for comprehensive functional analysis, paving the way for the discovery of gene regulation, disease mechanisms and potential therapeutic interventions in *C. elegans* and beyond.

## Data Availability

The original contributions presented in the study are included in the article/Supplementary Material, further inquiries can be directed to the corresponding author.

## References

[B1] Chavez A. Tuttle M. Pruitt B. W. Ewen-Campen B. Chari R. Ter-Ovanesyan D. (2016). Comparison of Cas9 activators in multiple species. Nat. Methods 13, 563–567. 10.1038/nmeth.3871 27214048 PMC4927356

[B2] Chen R. A. Down T. A. Stempor P. Chen Q. B. Egelhofer T. A. Hillier L. W. (2013). The landscape of RNA polymerase II transcription initiation in *C. elegans* reveals promoter and enhancer architectures. Genome Res. 23, 1339–1347. 10.1101/gr.153668.112 23550086 PMC3730107

[B3] Clement K. Hsu J. Y. Canver M. C. Joung J. K. Pinello L. (2020). Technologies and computational analysis strategies for CRISPR applications. Mol. Cell. 79, 11–29. 10.1016/j.molcel.2020.06.012 32619467 PMC7497852

[B4] El-Brolosy M. A. Stainier D. Y. R. (2017). Genetic compensation: a phenomenon in search of mechanisms. PLoS Genet. 13, e1006780. 10.1371/journal.pgen.1006780 28704371 PMC5509088

[B5] Fischer F. Benner C. Goyala A. Grigolon G. Vitiello D. Wu J. (2022). Ingestion of single guide RNAs induces gene overexpression and extends lifespan in *Caenorhabditis elegans via* CRISPR activation. J. Biol. Chem. 298, 102085. 10.1016/j.jbc.2022.102085 35636511 PMC9243178

[B6] Gilbert L. A. Horlbeck M. A. Adamson B. Villalta J. E. Chen Y. Whitehead E. H. (2014). Genome-scale CRISPR-mediated control of gene repression and activation. Cell. 159, 647–661. 10.1016/j.cell.2014.09.029 25307932 PMC4253859

[B7] Kruesi W. S. Core L. J. Waters C. T. Lis J. T. Meyer B. J. (2013). Condensin controls recruitment of RNA polymerase II to achieve nematode X-chromosome dosage compensation. Elife 2, e00808. 10.7554/eLife.00808 23795297 PMC3687364

[B8] Li W. X. Li G. H. Tong X. Yang P. P. Huang J. F. Xu L. (2020). Systematic metabolic analysis of potential target, therapeutic drug, diagnostic method and animal model applicability in three neurodegenerative diseases. Aging (Albany NY) 12, 9882–9914. 10.18632/aging.103253 32461378 PMC7288927

[B9] Lindholm D. Wootz H. Korhonen L. (2006). ER stress and neurodegenerative diseases. Cell. Death Differ. 13, 385–392. 10.1038/sj.cdd.4401778 16397584

[B10] Maeder M. L. Linder S. J. Cascio V. M. Fu Y. Ho Q. H. Joung J. K. (2013). CRISPR RNA-Guided activation of endogenous human genes. Nat. Methods 10, 977–979. 10.1038/nmeth.2598 23892898 PMC3794058

[B11] Roux A. E. Yuan H. Podshivalova K. Hendrickson D. Kerr R. Kenyon C. (2023). Individual cell types in *C. elegans* age differently and activate distinct cell-protective responses. Cell. Rep. 42, 112902. 10.1016/j.celrep.2023.112902 37531250

[B12] Saito T. L. Hashimoto S. Gu S. G. Morton J. J. Stadler M. Blumenthal T. (2013). The transcription start site landscape of *C. elegans* . Genome Res. 23, 1348–1361. 10.1101/gr.151571.112 23636945 PMC3730108

[B13] Schweinsberg P. J. Grant B. D. (2013). *C. elegans* gene transformation by microparticle bombardment. WormBook, 1–10. 10.1895/wormbook.1.166.1 PMC4050979 24395815

[B14] Timmons L. Tabara H. Mello C. C. Fire A. Z. (2003). Inducible systemic RNA silencing in *Caenorhabditis elegans* . Mol. Biol. Cell. 14, 2972–2983. 10.1091/mbc.e03-01-0858 12857879 PMC165691

[B15] Wang W. Sun Y. Liu X. Kumar S. K. Jin F. Dai Y. (2022). Dual-targeted therapy circumvents non-genetic drug resistance to targeted therapy. Front. Oncol. 12, 859455. 10.3389/fonc.2022.859455 35574302 PMC9093074

[B16] Wang P. Cao Z. Wang Q. Ma X. Wang N. Chen L. (2023). Protocol for CRISPR-Cas9-mediated genome editing to study spermatogenesis in *Caenorhabditis elegans* . Star. Protoc. 4, 102720. 10.1016/j.xpro.2023.102720 37967017 PMC10684875

[B17] Zou L. Wu D. Zang X. Wang Z. Wu Z. Chen D. (2019). Construction of a germline-specific RNAi tool in *C. elegans* . Sci. Rep. 9, 2354. 10.1038/s41598-019-38950-8 30787374 PMC6382888

